# The Current Landscape of Immune Checkpoint Blockade in Metastatic Lung Squamous Cell Carcinoma

**DOI:** 10.3390/molecules26051392

**Published:** 2021-03-05

**Authors:** Hong Yuan, Jing Liu, Jun Zhang

**Affiliations:** 1 Department of Oncology, Ruijin Hospital, Shanghai Jiao Tong University School of Medicine, Shanghai 200025, China; yh12551@rjh.com.cn; 2 State Key Laboratory of Oncogenes and Related Genes, Shanghai Jiao Tong University School of Medicine, Shanghai 200025, China

**Keywords:** lung squamous cell carcinoma, immunotherapy, immune checkpoint blockade, tumor microenvironment

## Abstract

In addition to surgery, chemotherapy, radiotherapy, and targeted therapy, immunotherapy has emerged as a standard pillar of cancer treatment. Immune checkpoint inhibitors (ICIs) such as targeting programmed death-1/programmed death ligand 1 (PD-1/PD-L1) and cytotoxic T lymphocyte antigen 4 (CTLA-4) have been integrated into standard-of-care regimens for patients with advanced lung squamous cell carcinoma (LUSC), who were previously limited by the lack of treatment options. Atezolizumab, durvalumab, nivolumab, and pembrolizumab are all currently used as part of standard-of-care treatment for different stages of lung cancer. Recent successes and failures of immune checkpoint blockade-based combination therapies have provided significant insights into implementing combination strategies in LUSC. Therefore, there is an urgent need to correctly select patients who are more likely to respond to immunotherapy and understand the mechanisms of primary or acquired resistance. In this review, we aim at summarizing the emerging clinical data on the promise and challenge of ICIs, discussing the unmet need of potential biomarkers for predicting response or resistance to immunotherapy, and providing an overview of the current immune landscape and future directions in advanced LUSC.

## 1. Introduction

Lung cancer is the leading cause of cancer-related mortality all over the world [1,2]. Lung squamous cell carcinoma (LUSC), a subtype of non-small cell lung cancer (NSCLC), comprises approximately 30% of NSCLC and is attributable to smoking. Development of squamous cell carcinoma (SCC) is related to genomic perturbations, gene mutations, chromosomal instabilities, and/or altered expression of key molecules involved in different stages of squamous cell lineage commitment and/or terminal differentiation.

Patients with driver mutation positive lung adenocarcinoma (LUAD) can benefit from a targeted therapy such as tyrosine kinase inhibitors (TKIs), while few druggable targets have been identified in LUSC [3,4]. Standard platinum-based doublet chemotherapy has always represented the first-line therapy for advanced/metastatic LUSC [5,6].

The FLEX trial showed that overall survival (OS) was prolonged in a chemotherapy plus cetuximab group (11.3 months) versus a chemotherapy alone group (10.1 months) in patients with advanced NSCLC across all histological subtypes (adenocarcinoma, squamous cell carcinoma, etc.) [7]. The SQUIRE trial demonstrated that the addition of necitumumab, a second-generation EGFR antibody, to platinum-based doublet chemotherapy improved survival (mOS, 11.5 months versus 9.9 months) in patients with previously untreated stage IV squamous NSCLC [8]. However, the survival benefit from the combination of chemotherapy and targeted therapy was limited for advanced LUSC patients.

Lee J.S. indicated that anti-programmed death-1/programmed death ligand 1 (anti-PD-1/PD-L1) therapy can provide significant survival benefits and a high objective response rate (ORR) for LUSC patients [9]. The emergence of immune checkpoint inhibitors (ICIs) has revolutionized the standard care of patients with LUSC, previously limited by the lack of treatment selections [10]. Motivated by this progress, a large number of immunotherapy-based studies have currently been implemented worldwide, alone or in combination with other systemic therapies.

In this review, we summarize PD-1/PD-L1 inhibitors in clinical practice (Table 1), recent clinical trials on ICIs in LUSC, and discuss the need for biomarkers for predicting potential response or resistance to immunotherapies.

## 2. Immune Checkpoint Inhibitors (ICIs) in Advanced Lung Squamous Cell Carcinoma (LUSC) after First-Line Therapy Failure

Immunotherapy is widely used in the care of patients with NSCLC, and multiple standard-of-care (SOC) regimens are approved in metastatic LUSC (Table 2). Studies such as KeyNote-010 [11], CheckMate 017 [12,13,14] /078 [15], POPLAR [16], and OAK [17,18] have proven that ICIs can improve the prognosis of patients with advanced NSCLC, bringing about a new alternative for second-line treatment.

Studies have evaluated single agent PD-1/PD-L1 blockade in previously treated, advanced NSCLC and have demonstrated improved efficacy over standard chemotherapy with docetaxel. Nivolumab, pembrolizumab, and atezolizumab were approved based on the results of CheckMate 017 [12], KeyNote-010 [11], and OAK [17,18], respectively. Compared with that in chemotherapy, the hazard ratio (HR) for the OS of ICIs ranged from 0.59 to 0.73. Importantly, the significant improvement in the OS came with notable reductions in toxicity. The incidence of severe or life-threatening adverse events (grade 3 or higher) ranged from 7% to 15% with ICIs versus 35% to 55% as compared with docetaxel.

## 3. ICIs in Advanced LUSC in First-Line Treatment

Treatment with PD-1/PD-L1 blockade has now become the front-line therapy in patients with lung cancer (Table 3). In the KeyNote-024 study [19], patients with PD-L1 high expression, tumor proportion score (TPS) > 50%, and without targetable alterations in epidermal growth factor receptor (EGFR) or anaplastic lymphoma kinase (ALK) were randomized to receive either pembrolizumab or platinum-based doublet chemotherapy. The median progression-free survival (PFS) was significantly longer in the pembrolizumab group than that of standard platinum-based doublet chemotherapy (10.3 vs. 6 months, HR 0.50, 95% CI 0.37–0.68, *p* < 001). From longer term follow-up data, the median OS for the pembrolizumab group was 30 months as compared with 14.2 months in the chemotherapy group (HR 0.49, 95% CI 0.34–0.69).

For those patients who may not tolerate chemotherapy and have PD-L1 expression (TPS > 1%), single-agent pembrolizumab may be taken into consideration based on KeyNote-042 [20], although the benefit of this approach was largely driven by patients with high expression of PD-L1.

In KeyNote-407 study [21], patients were randomized to a chemotherapy backbone of carboplatin and investigator’s choice of either paclitaxel or nanoparticle-bound paclitaxel plus pembrolizumab or placebo. The median OS was 15.9 months with pembrolizumab plus chemotherapy versus 11.3 months with placebo plus chemotherapy (HR for death, 0.64, 95% CI 0.49–0.85, *p* < 001). This combination is applicable to patients with either squamous or non-squamous cell carcinoma regardless of PD-L1 expression level.

IMpower 131 study [22] revealed that atezolizumab in combination with carboplatin and nab-paclitaxel (A + CnP) as first-line treatment, significantly improved PFS as compared with chemotherapy alone in patients with stage IV squamous NSCLC, but the OS was similar among A + CP (atezolizumab+ carboplatin+ paclitaxel), A + CnP, and CnP (carboplatin + nab-paclitaxel) arms.

Front-line nivolumab plus ipilimumab was compared with chemotherapy in the CheckMate 227 trial [23,24,25] and the results indicated that chemotherapy free might be an option for patients who showed intolerance to chemotherapy and were not optimal candidates for single-agent anti-PD-1/PD-L1 therapy. This study enrolled patients with advanced, metastatic NSCLC stratified by PD-L1 expression ≥1% versus <1% and randomized 1:1:1 to nivolumab plus ipilimumab, nivolumab alone, or platinum doublet chemotherapy. An earlier report of the first co-primary endpoint of the study highlighted high tumor mutational burden (TMB) (≥10 mutations/Mb) as a biomarker predicting longer PFS with combination immunotherapy; however, this result did not persist in the OS analysis. In the second co-primary endpoint, across PD-L1 expression levels, the median OS was longer in the immunotherapy group than that in the chemotherapy group. Specifically, in patients with PD-L1 expression (TPS ≥ 1%), the median OS was 17.1 months (95% CI, 15.0–20.1 months) with nivolumab plus ipilimumab versus 14.9 months (95% CI, 12.7–16.7 months) with chemotherapy, whereas in the PD-L1 < 1% group, the median OS was 17.2 months (95% CI, 12.8–22.0 months) versus 12.2 months (95% CI, 9.2–14.3 months), respectively.

## 4. Immune Checkpoint Blockade-Based Combination Strategies

PD-L1 and programmed death ligand 2 (PD-L2) are ligands of PD-1 and the interaction of PD-L1 or PD-L2 with PD-1 results in T cell suppression. The affinity of PD-L2/PD-1 is 2~6-fold higher than the interaction of PD-L1/PD-1 [26]. Additionally, the binding of PD-L1 to the cluster of differentiation 80 (CD80) on activated T cells can inhibit T cell activity. Conversely, PD-L2 can interact with repulsive guidance molecule B (RGMB) receptor, a co-receptor for bone morphogenetic protein (BMP), resulting in the activation of T cells [27].

PD-1 antibodies can block PD-1/PD-L1 and PD-1/PD-L2 pathways but leave the PD-L1/CD80 axis unaffected, leading to the inhibition of T cell activation. In addition, anti-PD-1 indirectly enhances PD-L2/RGMB interaction, resulting in the improvement of T cell-mediated anti-tumor immune response. Unlike the PD-1 antibodies, the PD-L1 antibodies also block the PD-1/PD-L1 and PD-L1/CD80 axes but leave the PD-L2/PD-1 axis unaffected, leading to tumor immune escape [28]. Such evolving knowledge of the biochemical and signaling effects will shed light on the molecular mechanisms of PD-1 checkpoint blockade-responsive patients and guide the design of combination therapies to modulate PD-1 and its downstream targets.

Immune checkpoint blockers activate effector T cells, which in turn facilitate the normalization of tumor blood vessels. The tumor vascular normalization promotes effector T cells infiltration into tumors and improves their function, leading to the enhancement of antitumor immunity [29]. This feedback loop between immune reprogramming and tumor vascular normalization reinforces itself, ultimately promoting immune-mediated tumor eradication [30]. The immune reprogramming-tumor vasculature crosstalk provides us with a rationale for combination approaches using immunotherapy and antiangiogenic therapy [31].

Clinical trials combining PD-1/PD-L1 blockade with cytotoxic therapy or with other ICI strategies have shown higher response rates at the risk of higher toxicity. According to the CheckMate 227 study, nivolumab plus ipilimumab was an option for first-line treatment of advanced NSCLC without driver mutations in patients with PD-L1 TPS ≥ 1%. However, there is no head-to-head comparison of anti-PD-1 plus chemotherapy versus anti-PD-L1 plus chemotherapy for advanced LUSC patients. A meta-analysis using data from the KeyNote-407 and IMpower 131 studies revealed that pembrolizumab brought significantly better OS (HR 0.67, *p* = 0.02) and numerically superior PFS (HR 0.79, *p* = 0.10) as compared with atezolizumab in LUSC patients [32]. Therefore, studies have been implemented to explore the optimal choice of immunotherapy (anti-PD-1, anti-PD-L1, or anti-CTLA-4) for advanced LUSC patients in combination with chemotherapy [33,34,35].

## 5. Biomarkers Predicting the Efficacy of Immunotherapy

Only a minority of patients have achieved sustained benefits from ICI; reliable biomarkers that can accurately identify these patients before or early during treatment have thus far remained elusive. Biomarkers such as PD-L1 (Table 4), TMB (Table 5), or tumor inflammation, offer insight into which patients may benefit most from the emerging agents and combination strategies. PD-L1 has several major shortcomings as a predictive biomarker of durable benefit while TMB is continuing to be evaluated clinically.

The current practice in the USA for front-line therapy of driver mutation-negative advanced NSCLC is to treat patients with PD-L1 ≤ 49% (percentage of PD-L1 positive tumor cells by immunohistochemistry) with concurrent chemotherapy plus pembrolizumab, and those with PD-L1 ≥ 50% with pembrolizumab alone or with concurrent chemotherapy.

Studies have shown that ICIs improve overall survival as compared with chemotherapy in first-line therapy for patients with PD-L1 expression ≥ 50%. A combination of ICIs with chemotherapy improves survival in patients with both squamous and non-squamous NSCLC, regardless of the expression level of PD-L1. However, a subset of patients with PD-L1 TPS < 1% can benefit from immunotherapy alone [12], suggesting that PD-L1 is an imperfect predictive biomarker. PD-L1 immunohistochemistry (IHC) staining alone is not sufficiently accurate to identify potential responders to PD-1/PD-L1 blockade-based immunotherapy in NSCLC [36,37]. In addition, heterogeneity within tumors, biopsies from primary versus metastatic lesions, different detection antibodies and cut-offs, staining procedures, and immune escape to PD-L1/PD-1 blockade are key factors that likely relate to the predictive value of PD-L1 [38,39,40]. One approach to resolve the limitation of spatial resolution involves positron emission tomography (PET)-based PD-L1 imaging with zirconium-89-labeled atezolizumab [41,42]. The technique, a noninvasive imaging of tumor PD-L1 expression in vivo, may enable patient selection from anti-PD-1/PD-L1 therapy and monitor PD-L1 expression during therapy [41]. Bensch F. et al. presented the initial results from a first-in-human study to assess the feasibility of imaging with zirconium-89-labeled atezolizumab and tested its potential to predict a clinical response to PD-L1 blockade in NSCLC [42].

CheckMate 227 (nivolumab plus ipilimumab versus platinum-doublet chemotherapy as first-line therapy for advanced NSCLC) displayed that TMB correlated with the clinical response to combination therapy with nivolumab and ipilimumab as a first-line regimen for NSCLC [23]. The CheckMate 227 trial was the first study that evaluated TMB as a biomarker in NSCLC patients who received immunotherapy. A significant benefit such as prolonged PFS was not observed in the overall population who received the combination therapy as compared with chemotherapy alone. Patients with TMB ≥ 10 mutations/Mb who received the combination treatment showed significantly better PFS as compared with those who received chemotherapy alone, irrespective of the expression of PD-L1. TMB might be a predictive biomarker for NSCLC patients who would benefit from ICB-based therapy [43].

In the future, tumor biopsy tissue collected prior to treatment, on-treatment, and disease progression will likely enable greater sophistication in treatment selection and achieve immune eradication of tumors.

## 6. Strategies Overcoming Immunotherapy Resistance

The biology underlying resistance is driven by the interplay between the immune system and tumor microenvironment (TME) and is influenced by multiple factors. Patients who have disease progression after receiving at least 6 weeks of exposure to PD-1/PD-L1 checkpoint inhibitors, but with PD/SD for ≤6 months can be considered to have primary resistant disease. Patients with SD for ≥6 months, who then had overall disease progression, independent of the target lesion measurements (overall tumor regression of <30% or tumor growth <20%), would be considered to have secondary resistance [44].

ICIs-targeted regimens can fail patients because of primary resistance, when a cancer does not respond to immunotherapy, or acquired resistance, i.e., when a cancer initially responds but then progresses [44,45]. Constitutive mechanisms of resistance to ICB therapies include innate PD-1 resistance signature (IPRES), oncogene activation (Wnt/β-catenin), loss of oncosuppressor genes (PTEN), downregulation of the type I IFN receptor IFNAR1 on cytotoxic T lymphocytes (CTLs), and poor infiltration by CD8^+^ T cells. Acquired resistance to ICIs includes disruption/downregulation of antigen presentation machinery, loss of IFN-γ sensitivity, neoantigen depletion, tumor-mediated immune suppression or immune exclusion, and additional inhibitory checkpoints [45].

Among tumor types, there appears to be an inverse association between overall response rate and the frequency of acquired resistance among responders to PD-1 blockade [46]. Strategies to combat resistance to immunotherapy involve enhancing antigenicity, modulation of TME, increasing immune cell activity, and overcoming resistance mediated by other upregulated immune checkpoints.

Mechanism-based strategies to overcome resistance to immune checkpoint targeted therapies (ICT) are urgently needed. Ishizuka J.J. et al. showed that loss of RNA-editing enzyme ADAR1 overcomes resistance to PD-1 checkpoint blockade caused by inactivation of antigen presentation in tumor cells [47]. Cortez M.A. et al. found that BMP7 impairs proinflammatory responses in the tumor microenvironment by inhibiting mitogen-activated protein kinase 14 (MAPK14) expression in macrophages and CD4^+^ T cells. Knockdown of BMP7 in combination with anti-PD1 activates CD4^+^ and CD8^+^ T cells in tumors, decreases M2 macrophages, and resensitizes resistant tumors to immunotherapies [48]. Emerging evidence demonstrates that targeting epigenetic elements that promote tumor progression and inhibit immune cell activity can enhance antitumor immunity by reshaping the TME. Enhancer of zeste homolog 2 (EZH2), the catalytic subunit of polycomb repressive complex 2 (PRC2), inhibition can increase T regulatory cell trafficking, impair T regulatory cell capacity, increase antigen presentation, and increase antitumor immunity, making it a promising target for overcoming ICB resistance of certain cancers [49].

The search for better prognostic and predictive biomarkers is ongoing and will be essential for improving patient selection for the growing list of therapeutic options [50].

## 7. Conclusions

In-depth tumor analysis including whole-genome sequencing, single cell RNA-sequencing, multidimensional flow cytometry, or epigenetics might be implemented in the future in order to find individualized treatment strategies.

Consideration of common properties across SCCs as they relate to epigenetics, genomics, genetics, and transcriptomes may serve as a foundation for individual and combinatorial therapeutics, as well as understanding the mechanistic basis of treatment resistance.

The emergence of PD-1/PD-L1/CTLA-4-targeted therapy has revolutionized the standard care of LUSC patients, generating durable responses and prolonged overall survival. However, not all patients benefit from immunotherapy. Primary or acquired resistance prevents patients from experiencing tumor shrinkage or extended survival. Predictive biomarkers such as PD-L1, TMB, or tumor inflammation, deserve further study.

An improved understanding of the biology and molecular subtypes of non-small cell lung cancer has resulted in the development of new biomarker-directed therapies and has led to improvements in overall survival of patients with advanced or metastatic disease. Since the introduction of PD-1/PD-L1 blockers in 2015, patients without molecular therapeutic targets now receive treatment with one of the immune checkpoint therapies in the first-line setting, especially for squamous NSCLC patients. Compared with previous studies, we have focused on the landscape of ICB in metastatic lung squamous cell carcinoma [43,51,52].

In conclusion, immunotherapy has changed the SOC for NSCLC, and has paved the way for a new treatment paradigm. Despite these advances, monumental efforts are still needed to significantly improve the outcome of patients with advanced/metastatic LUSC.

## Figures and Tables

**Table 1:** Programmed death-1/programmed death ligand 1 (PD-1/PD-L1) inhibitors were used in clinical practice for lung squamous cell carcinoma (LUSC) patients.

Drug	Target	FDA Indication	EMA Indication	NMPA Indication
Pembrolizumab (Keytruda)	PD-1	Pembrolizumab plus standard chemotherapy (carboplatin and either paclitaxel or nab-paclitaxel) for first-line treatment in patients with metastatic LUSC; Approved for treatment of advanced or metastatic NSCLC patients with disease progression after platinum-based chemotherapy and PD-L1 positive	Approved for first-line treatment of metastatic NSCLC patients with PD-L1 TPS ≥ 50%, without a sensitizing EGFR mutation or ALK translocation; Approved for treatment of locally advanced or metastatic NSCLC patients with PD-L1 TPS ≥ 1%, and disease progression during/after first-line chemotherapy	Approved for first-line treatment of locally advanced or metastatic NSCLC patients with PD-L1 TPS ≥ 1%, without a sensitizing EGFR mutation or ALK translocation; Pembrolizumab plus standard chemotherapy (carboplatin and either paclitaxel or nab-paclitaxel) for first-line treatment in patients with metastatic LUSC
Nivolumab (Opdivo)	PD-1	Approved for treatment of patients with previously treated advanced NSCLC whose disease progression during/after platinum-based doublet chemotherapy	Approved for treatment of patients with locally advanced or metastatic NSCLC whose disease progression during/after chemotherapy	Approved for second-line or late-line treatment of patients with locally advanced or metastatic NSCLC without a sensitizing EGFR mutation or ALK translocation, and disease progression during/after platinum-based doublet chemotherapy
Tislelizumab (TIZLEIO)	PD-1	NA	NA	Tislelizumab plus standard chemotherapy (carboplatin and paclitaxel) for first-line treatment of locally advanced or metastatic LUSC patients
Atezolizumab (Tecentriq)	PD-L1	Approved for treatment of patients with metastatic NSCLC whose disease progression during or after platinum-based chemotherapy	Approved for treatment of patients with locally advanced or metastatic NSCLC whose disease progression during/after chemotherapy	NA

Abbreviations: PD-1, programmed death-1; PD-L1, programmed death ligand 1; LUSC, lung squamous cell carcinoma; FDA, Food and Drug Administration; EMA, European Medicines Agency; NMPA, National Medical Products Administration; NSCLC, non-small cell lung cancer; TPS, tumor proportion score; EGFR, epidermal growth factor receptor; ALK, anaplastic lymphoma kinase; NA, not available.

**Table 2:** The second-line clinical trials of anti-PD-1/PD-L1 in metastatic LUSC.

Trials	CheckMate 017	CheckMate 078	KeyNote 010	POPLAR	OAK
Histology	LUSC	NSCLC	NSCLC (TPS ≥ 1%)	NSCLC	NSCLC
Treatment Comparison	Nivolumab Docetaxel	Divolumab Docetaxel	Pembrolizumab Docetaxel	Atezolizumab Docetaxel	Atezolizumab Docetaxel
ORR	20% vs. 8%	16.6% vs. 4.2%	19% vs. 10%	NA	14% vs. 13%
mOS Squamous Non-squamous	9.2 m vs. 6.0 m / /	12.0 m vs. 9.6 m 12.3 m vs. 7.9 m 11.9 m vs. 10.2 m	12.7 m vs. 10.4 m / /	12.6 m vs. 9.7 m 10.1 m vs. 8.6 m 15.5 m vs. 10.9 m	13.8 m vs. 9.6 m 8.9 m vs. 7.7 m 15.6 m vs. 11.2 m
OS HR	0.59 (0.44–0.79)	0.68 (0.52–0.90)	0.71 (0.58–0.88)	0.73 (0.53–0.99)	0.73 (0.62–0.87)

Abbreviations: PD-1, programmed death-1; PD-L1, programmed death ligand 1; LUSC, lung squamous cell carcinoma; NSCLC, non-small cell lung cancer; TPS, tumor proportion score; ORR, objective response rate; mOS, median overall survival; HR, hazard ratio.

**Table 3:** The first-line clinical trials of immune checkpoint blockers in advanced LUSC.

Trials	ORIENT-12		RATIONALE 307			KeyNote-407		IMpower 131	
Histology	LUSC		LUSC			LUSC		LUSC	
Group	Experimental Group	Control Group	Experimental Group A	Experimental Group B	Control Group	Experimental Group	Control Group	Experimental Group	Control Group
N	179	178	120	119	121	278	281	343	340
Treatment	Sintilimab gemcitabine cisplatin/ carboplatin	Placebo gemcitabine cisplatin/ carboplatin	Tislelizumab carboplatin paclitaxel	Tislelizumab carboplatin nab-paclitaxel	Carboplatin paclitaxel	Pembrolizumab carboplatin paclitaxel/ nab-paclitaxel	Placebo carboplatin paclitaxel/ nab-paclitaxel	Atezolizumab carboplatin nab-paclitaxel	Carboplatin nab-paclitaxel
ORR	44.7%	35.4%	73.0%	75.0%	50.0%	62.6%	38.4%	49.7%	41.0%
mDoR	/	/	8.2 m	8.6 m	4.2 m	8.8 m	4.9 m	7.3 m	5.2 m
mPFS	5.5 m	4.9 m	7.6 m	7.6 m	5.5 m	6.4 m	4.8 m	6.3 m	5.6 m
PFS HR	0.54		0.52	0.48	/	0.56		0.71	
mOS	NA	NA	NA	NA	NA	15.9 m	11.3 m	14.2 m	13.5 m
OS HR	0.57		/			0.64		0.88	

Abbreviations: LUSC, lung squamous cell carcinoma; ORR, objective response rate; mDoR, median duration of response; mPFS, median progression-free survival; HR, hazard ratio; mOS, median overall survival; NA, not available.

**Table 4:** Summary of PD-L1 immunohistochemistry assays in non-small cell lung cancer (NSCLC).

Antibody Clone	22C3	28-8	SP142	SP263
Species of origin	Mouse	Rabbit	Rabbit	Rabbit
Instrument	Dako Autostainer Link48	Dako Autostainer Link48	Ventana Benchmark ULTRA	Ventana Benchmark ULTRA
Detection system	EnVision Flex	EnVision Flex	Optiview and Amplification	Optiview
Targeted cell	TC	TC	TC/IC	IC
PD-L1 cutoff	TC ≥ 1%	TC ≥ 1%	TC ≥ 50%/IC ≥ 10%	TC ≥ 1%
Indication	Pembrolizumab CDx	Nivolumab complementary diagnostics	Atezolizumab CDx	Pembrolizumab CDx, Nivolumab complementary diagnostics

Abbreviations: PD-L1, programmed death-ligand 1; NSCLC, non-small cell lung cancer; TC, tumor cell; IC, immune cell; CDx, companion diagnostics.

**Table 5:** Overview of published studies assessing tumor mutational burden (TMB) in LUSC.

Cancer Type	NSCLC (*n* = 36)	Stage IV or Recurrent NSCLC (*n* = 312)	Stage IV or Recurrent LUSC (*n* = 100)
Clinical trial/drug	Anti-PD-1/PD-L1 monotherapy, anti-CTLA-4/anti-PD-1/PD-L1 combinations	CheckMate 026/nivolumab or platinum-based chemotherapy	CheckMate227/nivolumab plus ipilimumab, nivolumab plus chemotherapy, or chemotherapy
Definition of TMB	The number of somatic mutations by NGS	The total number of somatic missense mutations present in a baseline tumor sample	NA
Detection method of TMB	FoundationOne assay	Whole-exome sequencing	FoundationOne CDx assay
Cut-off value of TMB	≥20 mutations/Mb	≥243 mutations	≥10 mutations/Mb
Type of benefit	RR, PFS, OS	PFS, no benefit of OS	ORR, PFS

Abbreviations: TMB, tumor mutational burden; LUSC, lung squamous cell carcinoma; NSCLC, non-small cell lung cancer; PD-1, programmed death-1; PD-L1, programmed death ligand 1; CTLA-4, cytotoxic T lymphocyte antigen 4; NGS, next-generation sequencing; NA, not available; RR, response rate; PFS, progression-free survival; OS, overall survival; ORR, objective response rate.

## Data Availability

Not applicable.
